# Age, Body Mass Index, Tumor Subtype, and Racial and Ethnic Disparities in Breast Cancer Survival

**DOI:** 10.1001/jamanetworkopen.2023.39584

**Published:** 2023-10-25

**Authors:** Marla Lipsyc-Sharf, Karla V. Ballman, Jordan D. Campbell, Hyman B. Muss, Edith A. Perez, Lawrence N. Shulman, Lisa A. Carey, Ann H. Partridge, Erica T. Warner

**Affiliations:** 1 Department of Medical Oncology, Dana-Farber Cancer Institute, Boston, Massachusetts; 2 Harvard Medical School, Boston, Massachusetts; 3 David Geffen School of Medicine at UCLA/Jonsson Comprehensive Cancer Center, Los Angeles, California; 4 Alliance Statistics and Data Management Center, Mayo Clinic, Rochester, Minnesota; 5 University of North Carolina Lineberger Comprehensive Cancer Center, Chapel Hill; 6 Mayo Clinic Cancer Center, Jacksonville, Florida; 7 Abramson Cancer Center, University of Pennsylvania, Philadelphia; 8 Clinical Translational Epidemiology Unit, Mongan Institute, Massachusetts General Hospital, Boston

## Abstract

**Importance:**

Black women in the United States have higher breast cancer (BC) mortality rates than White women. The combined role of multiple factors, including body mass index (BMI), age, and tumor subtype, remains unclear.

**Objective:**

To assess the association of race and ethnicity with survival among clinical trial participants with early-stage BC (eBC) according to tumor subtype, age, and BMI.

**Design, Setting, and Participants:**

This cohort study analyzed survival data, as of November 12, 2021, from participants enrolled between 1997 and 2010 in 4 randomized adjuvant chemotherapy trials: Cancer and Leukemia Group B (CALGB) 9741, 49907, and 40101 as well as North Central Cancer Treatment Group (NCCTG) N9831, legacy groups of the Alliance of Clinical Trials in Oncology. Median follow-up was 9.8 years.

**Exposures:**

Non-Hispanic Black and Hispanic participants were compared with non-Hispanic White participants within subgroups of subtype (hormone receptor positive [HR+]/*ERBB2 *[formerly *HER2*] negative [*ERBB2*−], *ERBB2*+, and HR−/*ERBB2*−), age (<50, 50 to <65, and ≥65 years), and BMI (<18.5, 18.5 to <25.0, 25.0 to <30.0, and ≥30.0).

**Main Outcomes and Measures:**

Recurrence-free survival (RFS) and overall survival (OS).

**Results:**

Of 9479 participants, 436 (4.4%) were Hispanic, 871 (8.8%) non-Hispanic Black, and 7889 (79.5%) non-Hispanic White. The median (range) age was 52 (19.0-89.7) years. Among participants with HR+/*ERBB2*− tumors, non-Hispanic Black individuals had worse RFS (hazard ratio [HR], 1.49; 95% CI, 1.04-2.12; 5-year RFS, 88.5% vs 93.2%) than non-Hispanic White individuals, although the global test for association of race and ethnicity with RFS was not significant within any tumor subtype. There were no OS differences by race and ethnicity in any subtype. Race and ethnicity were associated with OS in young participants (age <50 years; global *P* = .008); young non-Hispanic Black participants (HR, 1.34; 95% CI, 1.04-1.71; 5-year OS, 86.6% vs 92.0%) and Hispanic participants (HR, 1.62; 95% CI, 1.16-2.29; 5-year OS, 86.2% vs 92.0%) had worse OS than young non-Hispanic White participants. Race and ethnicity were associated with RFS in participants with BMIs of 25 to less than 30, with non-Hispanic Black participants having worse RFS (HR, 1.81; 95% CI, 1.23-2.68; 5-year RFS, 83.2% vs 87.3%) than non-Hispanic White participants.

**Conclusions and Relevance:**

In this cohort study, racial and ethnic survival disparities were identified in patients with eBC receiving standardized initial care, and potentially at-risk subgroups, for whom focused interventions may improve outcomes, were found.

## Key Points

**Question:**

Are there racial and ethnic disparities in survival among participants enrolled in clinical trials receiving standardized initial care for early-stage breast cancer?

**Findings:**

In this cohort study with 9479 participants, pooled survival data suggest that survival differences exist even within clinical trial participants receiving similar initial care. Subgroups, defined by tumor subtype, age, and/or body mass index, that may drive racial and ethnic disparities in survival were identified.

**Meaning:**

These findings suggest potential factors contributing to racial and ethnic disparities in survival of patients with breast cancer; it is critical to evaluate interventions for improvement.

## Teaser

This cohort study assesses the association of race and ethnicity with survival among clinical trial participants with early-stage breast cancer according to tumor subtype, age, and body mass index (BMI).

## Introduction

For several decades, non-Hispanic Black women have had substantially higher breast cancer (BC) mortality rates than non-Hispanic White women.^1,2,3,4,5,6,7^ Recent data from the Surveillance, Epidemiology, and End Results program show that between 2015 and 2019, Black women had 41% higher BC mortality compared with White women despite a 4% lower incidence of BC.^8^ The emergence and subsequent widening of this disparity over the past 40 years suggests that potentially modifiable and time-varying factors contribute.^1^ Such disparities are also known to exist among Hispanic women, although there is a relative dearth of literature studying this important population.^9,10,11^ While lack of quality and timely initial adjuvant treatment is an important contributor, prospective study of BC survival in patients receiving standardized adjuvant therapy, as in the clinical trial setting, can facilitate identification of other factors.^10,11,12^

A previous pooled analysis of SWOG clinical trials showed that, while no racial disparities were observed for most cancer types, non-Hispanic Black women with BC were more likely to die than non-Hispanic White women.^13^ While this study did not investigate potential contributing factors, subsequent studies have suggested that racial and ethnic differences in BC survival vary by tumor subtype.^14,15,16,17^ Age has also been associated with survival in patients with BC. Both younger and older age have been associated with differences in treatments and adherence as well as worse mortality.^18,19,20,21,22^ Body mass index (BMI; calculated as weight in kilograms divided by height in meters squared) has also been shown to be associated with BC survival, although the role of BMI in racial and ethnic survival disparities, particularly among groups that are underreported and underrepresented in clinical trials, such as Hispanic patients, merits additional study.^20,21,23,24,25,26^ In general, the extent of disparities within subgroups defined by of age, BMI, and tumor subtype are unclear yet likely are important in addressing BC. In this pooled analysis of 4 prospective adjuvant BC clinical trials, we assessed whether race and ethnicity were associated with recurrence-free survival (RFS) and overall survival (OS) among women enrolled in clinical trials for early-stage BC (eBC) according to tumor subtype, age, and BMI.^14,15,16,17,20,21,24,25^

## Methods

### Data and Patients

**Figure 1.**
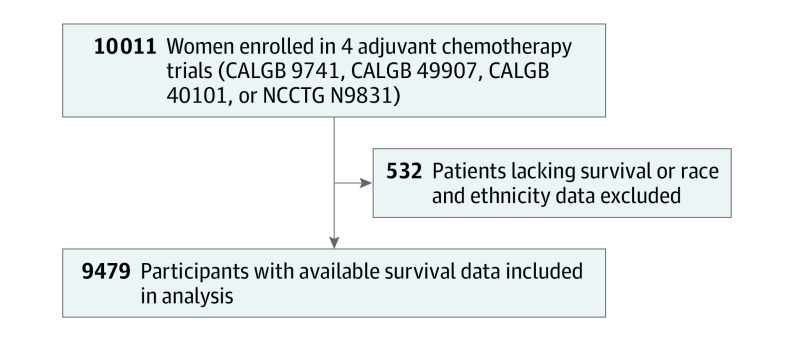
Flow Diagram of Participants CALGB indicates Cancer and Leukemia Group B; NCCTG, North Central Cancer Treatment Group.

We included participants enrolled in 4 adjuvant chemotherapy trials: Cancer and Leukemia Group B (CALGB) C9741 (NCT00003088), C49907 (NCT00005970), C40101 (NCT00024102) and North Central Cancer Treatment Group (NCCTG) N9831 (NCT00041119) (eMethods in Supplement 1).^27,28,29,30^ CALGB and NCCTG are now part of the Alliance for Clinical Trials in Oncology (Alliance). All participants with available survival and race and ethnicity data were included (Figure 1). Three of the trials (CALGB 9741, CALGB 49907, and CALGB 40101) evaluated adjuvant chemotherapy regimens for all BC subtypes, while NCCTG N9831 included only patients with human epidermal growth factor receptor 2–positive (*ERBB2*+) BC. CALGB 49907 studied older women with BC and included only patients ages 65 years and older. Relevant ethical review committees approved all trials. All participants in these trials provided informed consent. This study followed the Strengthening the Reporting of Observational Studies in Epidemiology (STROBE) reporting guideline.

### Measures and Outcomes

Tumor hormone receptor (HR) and *ERBB2* status were assessed in each trial as previously published.^27,28,29,30^ In this study, we analyzed the following subtypes: hormone receptor–positive/*ERBB2*-negative (HR+/*ERBB2*−), *ERBB2*+, and HR-negative/*ERBB2*-negative (HR−/ERBB2−; also known as triple negative). Race and ethnicity were analyzed in 4 groups: Hispanic, non-Hispanic Black, non-Hispanic White, and other race and ethnicity (including American Indian or Alaska Native, Asian, Native Hawaiian or Other Pacific Islander). The other race and ethnicity group was created because the sample sizes of the individual groups were too small to analyze separately. Race and ethnicity were patient-reported based on investigator options in 2 of the 4 trials included. For the other 2 trials, documentation of how race and ethnicity were collected is not available. Age at diagnosis was divided into 3 groups: younger than 50 years, 50 to younger than 65 years, and 65 years or older. Participant BMI at time of trial randomization was separated into 4 groups: less than 18.5, 18.5 to less than 25.0, 25.0 to less than 30.0, and 30.0 or greater, corresponding to the US Center for Disease Control and Prevention definitions of underweight, healthy weight, overweight, and obesity, respectively.^31^

RFS events included local, regional, or distant BC recurrence or death due to any cause.^32^ Participants alive without an RFS event were censored at the time of last follow-up. OS was defined using deaths from any cause as an event. Participants without known death were censored at the time of last contact, including any participants lost to follow-up. All time-to-event measures started at the time of trial randomization.

### Statistical Analysis

Median follow-up was estimated using the reverse Kaplan-Meier method.^33,34,35^ Baseline characteristics between race and ethnicity groups were compared using a χ^2^ test.^36^ All results presented are multivariable analyses. Cox proportional hazards models were used to estimate multivariable-adjusted hazard ratios (HRs) and 95% CIs for the association between race and ethnicity and RFS or OS.^34,35^ Multivariable models included race and ethnicity, tumor subtype, BMI, age, stage, and treatment group if the variable was not the subgroup being evaluated. Participants with missing data for subtype or stage were not included in the multivariable analyses controlling for subtype or stage, respectively. We also conducted separate analyses within strata of age and strata of BMI. Participants with missing data for BMI were not included in analyses within strata of BMI. No participants had missing age data. *P* values are provided only for global tests and not for comparisons of different levels of the variable with the reference group. We present point estimates and 95% CIs comparing each level of the variable with the reference group. Results of analyses were also depicted with forest plots.^37^ Kaplan-Meier estimators were used to estimate survival at specific time points and to generate survival curves.^34,35^ The prespecified level of significance was .05. The Alliance Statistics and Data Management Center conducted statistical analyses on the study database frozen on November 12, 2021, using SAS version 9.3 (SAS Institute).

## Results

### Patient Characteristics

Of 10 011 women enrolled in the included trials, 9479 (94.7%) had available survival and race and ethnicity data and were included in this pooled analysis. Their median (IQR) follow-up time was 9.8 (6.7-13.2) years, and 435 participants (4.6%) were designated as lost to follow-up. All participants were female. There were 436 (4.6%) Hispanic, 871 (9.2%) non-Hispanic Black, 7889 (83.2%) non-Hispanic White participants, and 283 (3.0%) patients with another race and ethnicity (Table). At enrollment, median (range) age was 52 (19.0-89.7) years, and median (range) BMI was 28.3 (12.3-77.3). The distribution of tumor subtypes differed significantly among race and ethnicity groups, with a larger proportion of non-Hispanic Black participants with HR−/*ERBB2*− BC compared with non-Hispanic White participants (197 [25.6%] vs 1049 [14.7%]; *P* < .001). Additionally, BMI differed significantly across race and ethnicity groups; obesity was more frequent in non-Hispanic Black participants than non-Hispanic White participants (511 [59.5%] vs 3008 [38.7%]; *P* < .001). The baseline characteristics between Hispanic and non-Hispanic White participants were similar.

**Table.** Participant Baseline Characteristics

Characteristic	Participants, No. (%)				
	Hispanic (n = 436)	Non-Hispanic Black (n = 871)	Non-Hispanic White (n = 7889)	Non-Hispanic other (n = 283)^a^	Total (N = 9479)
Trial					
CALGB 40101	206 (47.2)	357 (41.0)	2865 (36.3)	95 (33.6)	3523 (37.2)
CALGB 49907	30 (6.9)	59 (6.8)	460 (5.8)	10 (3.5)	559 (5.9)
CALGB 9741	80 (18.3)	216 (24.8)	1625 (20.6)	39 (13.8)	1960 (20.7)
NCCTG N9831	120 (27.5)	239 (27.4)	2939 (37.3)	139 (49.1)	3437 (36.3)
Age category, y					
<50	212 (48.6)	424 (48.7)	3285 (41.6)	138 (48.8)	4059 (42.8)
50 to <65	162 (37.2)	335 (38.5)	3385 (42.9)	109 (38.5)	3991 (42.1)
≥65	62 (14.2)	112 (12.9)	1219 (15.5)	36 (12.7)	1429 (15.1)
Stage					
I	113 (26.2)	177 (20.5)	1798 (22.9)	52 (18.7)	2140 (22.7)
II	268 (62.2)	533 (61.8)	4823 (61.5)	186 (66.9)	5810 (61.7)
III	50 (11.6)	152 (17.6)	1225 (15.6)	40 (14.4)	1467 (15.6)
Missing	5	9	43	5	62
Breast cancer subtype					
*ERBB2*+	172 (43.4)	320 (41.6)	3376 (47.3)	152 (59.1)	4020 (47.0)
HR+/*ERBB2*−	141 (35.6)	252 (32.8)	2711 (38.0)	72 (28.0)	3176 (37.1)
HR−/*ERBB2*−	83 (21.0)	197 (25.6)	1049 (14.7)	33 (12.8)	1362 (15.9)
Missing	40	102	753	26	921
BMI category^b^					
Underweight	4 (0.9)	8 (0.9)	129 (1.7)	5 (1.8)	146 (1.6)
Healthy weight	92 (21.3)	112 (13.0)	2315 (29.8)	115 (40.8)	2634 (28.2)
Overweight	146 (33.9)	228 (26.5)	2328 (29.9)	96 (34.0)	2798 (29.9)
Obesity	189 (43.9)	511 (59.5)	3008 (38.7)	66 (23.4)	3774 (40.4)
Missing	5	12	109	1	127

Abbreviations: −, negative; +, positive; BMI, body mass index (calculated as weight in kilograms divided by height in meters squared); CALGB, Cancer and Leukemia Group B; HR, hormone receptor; NCCTG, North Central Cancer Treatment Group.

^a^ Other race and ethnicity includes American Indian or Alaska Native, Asian, Native Hawaiian or Other Pacific Islander.

^b^ BMI groups were determined based on Center for Disease Control and Prevention definitions of underweight, healthy weight, overweight, and obesity as follows: less than 18.5, 18.5 to less than 25.0, 25.0 to less than 30.0, and 30.0 or greater respectively.

### Survival and Race and Ethnicity

#### Survival Within Tumor Subtypes by Race and Ethnicity

**Figure 2.**
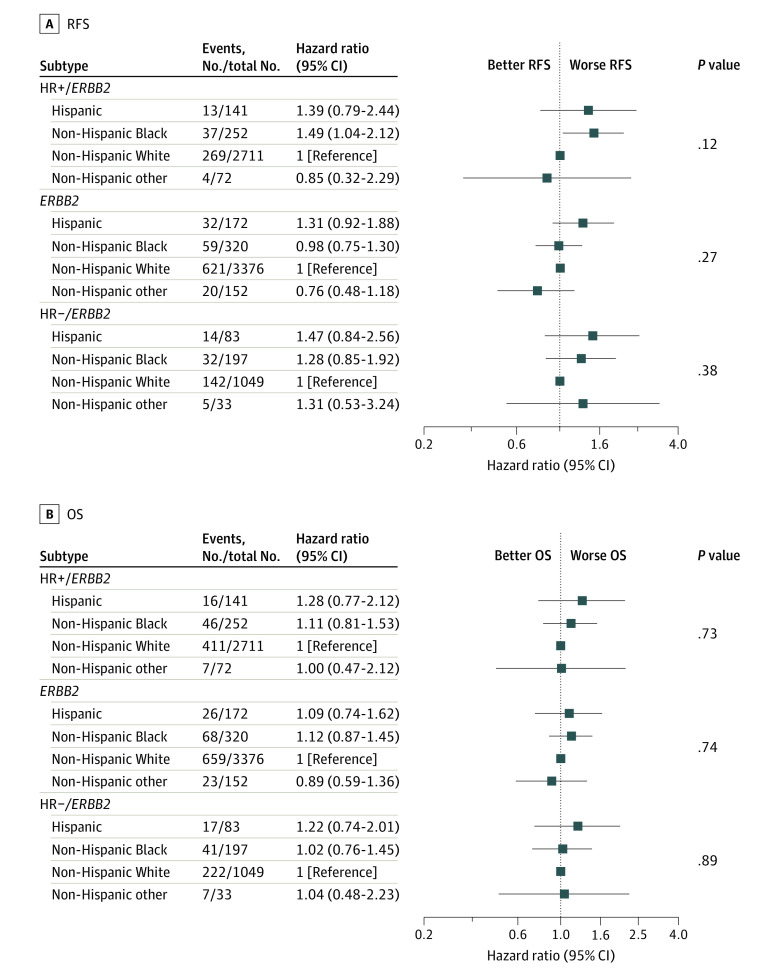
Forest Plots of Hazard Ratios Comparing Survival in Tumor Subtype by Race/Ethnicity − Indicates negative; +, positive; HR, hormone receptor; OS, overall survival; and RFS, recurrence-free survival.

We evaluated differences in RFS and OS among racial and ethnic groups for participants with the same BC subtype. Of all 9479 participants, 8588 (90.6%) had available tumor subtype data and were included in these analyses. Global tests for the association of the race and ethnicity variable with tumor subtype were not statistically significant within any subtype. However, we did observe an association among participants with HR+/*ERBB2*− tumors, with non-Hispanic Black individuals having worse RFS than non-Hispanic White individuals (HR, 1.49; 95% CI, 1.04-2.12; 5-year RFS, 88.5% vs 93.2%) (Figure 2). There were no differences in RFS observed between non-Hispanic Black and non-Hispanic White participants among those with *ERBB2*+ tumors (HR, 0.98; 95% CI, 0.75-1.30; 5-year RFS, 84.0% vs 85.1%) or HR−/*ERBB2*− tumors (HR, 1.28; 95% CI, 0.85-1.92; 5-year RFS, 84.1% vs 86.6%). There were no differences in RFS between Hispanic and non-Hispanic White participants, and no OS differences by race and ethnicity in any tumor subtype. Kaplan-Meier estimates of RFS and OS in BC subtype by race and ethnicity are depicted in eFigure 1 and eFigure 2 in Supplement 1.

#### Survival Within Age Categories by Race and Ethnicity

**Figure 3.**
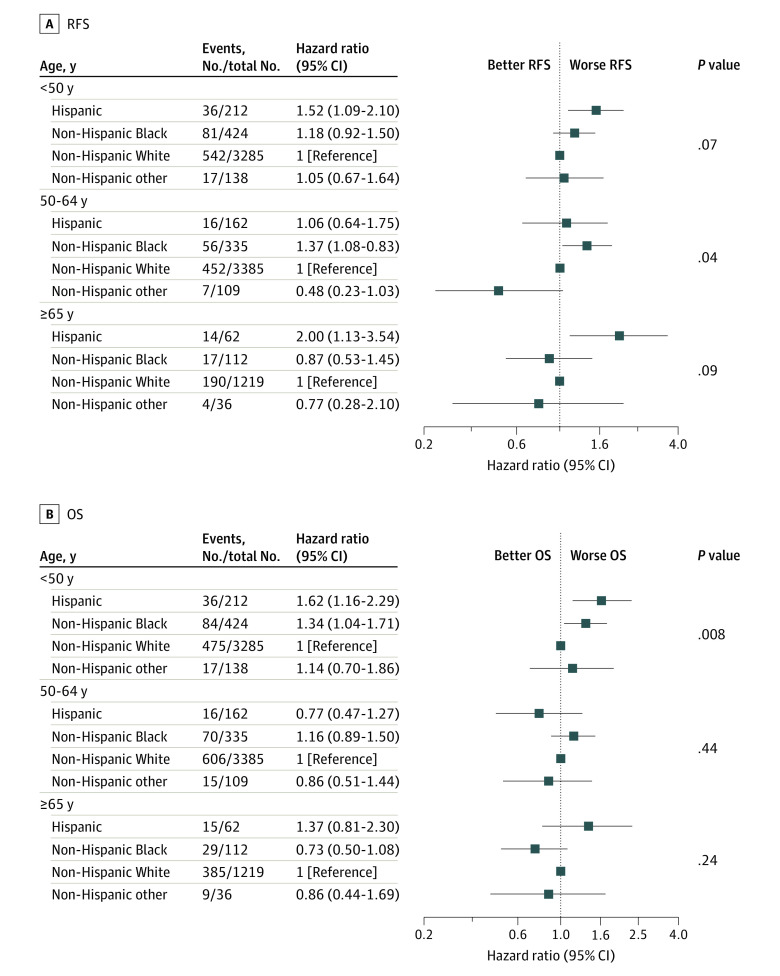
Forest Plots of Hazard Ratios Comparing Survival in Age Category by Race/Ethnicity OS indicates overall survival; RFS, recurrence-free survival.

We next evaluated survival differences within age groups. All 9479 participants had available age data and were included in these analyses. Within the middle age group, ages 50 to younger than 65 years, race and ethnicity were associated with RFS (global *P* = .04); non-Hispanic Black participants had worse RFS than non-Hispanic White participants (HR, 1.37; 95% CI, 1.03-1.83; 5-year RFS, 84.6% vs 89.1%) (Figure 3). Among younger (ages <50 years) and older (ages ≥65 years) patients, race and ethnicity were not significantly associated with RFS (global *P* = .07 and global *P* = .09, respectively). Both younger and older Hispanic participants had worse RFS than non-Hispanic White participants (younger: HR, 1.52; 95% CI, 1.09-2.10; 5-year RFS, 82.6% vs 85.9%; older: HR, 2.00; 95% CI, 1.13-3.54; 5-year RFS, 78.0% vs 87.9%). Race and ethnicity were significantly associated with OS in young participants (global *P* = .008). Specifically, young non-Hispanic Black (HR, 1.34; 95% CI, 1.04-1.71; 5-year OS, 86.6% vs 92.0%) and Hispanic (HR, 1.62; 95% CI, 1.16-2.29; 5-year OS, 86.2% vs 92.0%) participants had worse OS than young non-Hispanic White participants. There was no notable association observed between race and ethnicity and OS among participants ages 50 and older. Kaplan-Meier estimates of RFS and OS in age category by race and ethnicity are depicted in eFigure 3 and eFigure 4 in Supplement 1.

When further analyzed within subgroups jointly defined by subtype and age, race and ethnicity were associated with RFS in older participants with HR+/*ERBB2*− BCs (global *P* = .002). Among participants ages 65 years or older with HR+/*ERBB2*− tumors (n = 696), Hispanic participants (n = 23) had more than 6 times the risk of having an RFS event than non-Hispanic White participants (n = 537) (HR, 6.30; 95% CI, 2.41-16.50). Although the global test was not statistically significant for participants younger than 50 years with HR+/*ERBB2*− for RFS (*P* = .06) (n = 1288), non-Hispanic Black participants (n = 103) had considerably worse RFS (HR, 1.98; 95% CI, 1.15-3.42) than non-Hispanic White participants. This was also observed for OS with a non–statistically significant global test (*P* = .15) but with a considerably worse OS for non-Hispanic Black participants compared with non-Hispanic White participants (HR, 1.86; 95% CI, 1.08-3.18).

#### Survival Within BMI Categories by Race and Ethnicity

**Figure 4.**
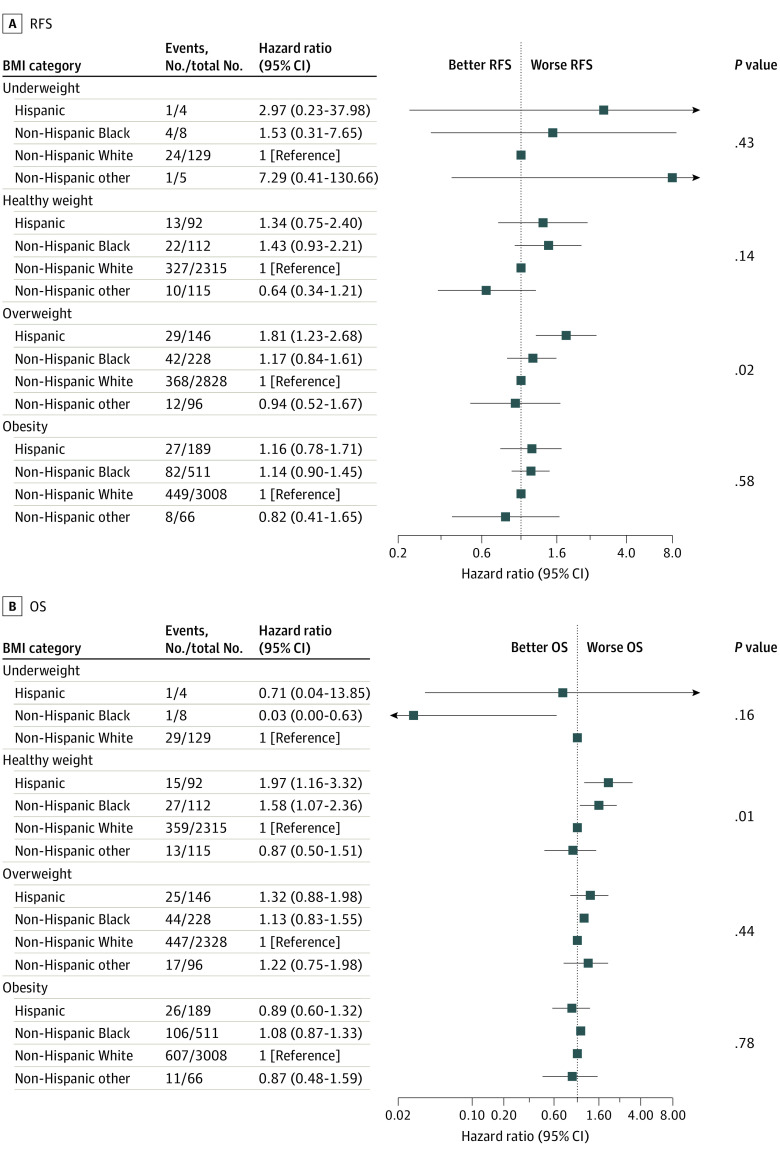
Forest Plots of Hazard Ratios Comparing Survival in Body Mass Index Category by Race/Ethnicity Body mass index (BMI; calculated as weight in kilograms divided by height in meters squared) groups were determined based on Center for Disease Control and Prevention definitions of underweight, healthy weight, overweight, and obesity as follows less than 18.5, 18.5 to less than 25.0, 25.0 to less than 30.0, and 30.0 or greater, respectively. Point estimates and 95% CIs for overall survival (OS) of participants in the non-Hispanic other race and ethnicity group with underweight could not be estimated. RFS indicates recurrence-free survival.

We also studied survival differences between race and ethnicity groups within BMI categories. Of all 9479 participants, 9352 (98.7%) had available BMI data and were included in these analyses. For participants with underweight and obesity, race and ethnicity were not significantly associated with RFS or OS on global testing. However, among participants with overweight, race and ethnicity were significantly associated with RFS (global *P* = .02), with Hispanic participants having worse RFS than non-Hispanic White participants (HR, 1.81; 95% CI, 1.23-2.68; 5-year RFS, 83.7% vs 87.3%) (Figure 4). For those with healthy weight, the association between race and ethnicity and RFS was not significant, although there was a significant association between race and ethnicity and OS (global *P* = .01): both non-Hispanic Black and Hispanic participants had worse OS than non-Hispanic White participants (non-Hispanic Black: HR, 1.58; 95% CI, 1.07-2.36; 5-year OS, 85.7% vs 91.5%; Hispanic: HR, 1.97; 95% CI, 1.16-3.32; 5-year OS, 85.0% vs 91.5%). Kaplan-Meier estimates of RFS and OS in BMI category by race and ethnicity are depicted in eFigure 5 and eFigure 6 in Supplement 1.

Analysis of subgroups jointly defined within BMI and subtype showed that, among patients with HR+/*ERBB2*− BC and overweight (n = 1054), non-Hispanic Black individuals (n = 69) had considerably worse RFS than non-Hispanic White individuals (n = 858) (HR, 2.12; 95% CI, 1.09-4.12), although the global test for race and ethnicity was not significant (global *P* = .08). This association was not statistically significant for OS. Within other subgroups defined by both BMI and subtype, there were no significant associations of race and ethnicity with RFS or OS.

## Discussion

We identified disease and patient subgroups with similarities and differences in survival among racial and ethnic groups for participants enrolled in 4 Alliance adjuvant chemotherapy clinical trials for eBC. While race and ethnicity, in general, were not associated with survival when stratified by subtype, non-Hispanic Black patients with HR+/*ERBB2*− tumors had worse RFS than non-Hispanic White patients. There were no OS differences by race and ethnicity in any subtype. We also observed survival differences within strata of some age groups: in middle-aged participants, non-Hispanic Black individuals had worse RFS than non-Hispanic White individuals. Among young participants, both non-Hispanic Black and Hispanic participants had worse OS than non-Hispanic White participants. Importantly, these survival differences existed even within clinical trial populations that ostensibly received standardized initial cancer care.

Prior work within the National Comprehensive Cancer Network Breast Cancer Outcomes Database and in the Carolina Breast Cancer Study demonstrated that racial disparities vary by tumor subtype, with differences in survival between non-Hispanic Black and non-Hispanic White patients with HR+ BC and no differences for patients with triple negative or *ERBB2*+ BC.^17,38^ However, these patients were not exclusively clinical trial participants. In contrast, there have been other analyses of survival differences in HR+ BCs within adjuvant clinical trials; however, unlike the present study, Hispanic patients and other race and ethnicity groups besides non-Hispanic Black and non-Hispanic White have not been specifically examined in these studies.^39,40^ We would advocate for the dedicated study of these patients in gaining understanding of disparities facing patients with HR+ BC and have included these populations in our analyses.

There are multiple plausible explanations for the differences observed between non-Hispanic Black and non-Hispanic White participants with HR+/*ERBB2*− BC in other studies and suggested in our study.^41^ Black patients are less likely to receive endocrine therapy, and some observational data suggests lower rates of adherence to endocrine therapy.^40,42,43^ Similarly, Black patients with HR+ BC are reported to have lower estrogen receptor staining levels.^44^ To better understand and address these factors, some investigators call for further biological delineation of HR+ subtype, evaluation of social determinants of health, and investigation into ancestry and country of origin in future studies.^41,45^

We found that, overall, young non-Hispanic Black and Hispanic patients have worse OS than young non-Hispanic White patients. It is reported that non-Hispanic Black and Hispanic women are more likely to be diagnosed with BC at a young age compared with non-Hispanic White women.^7,46^ While we identified differences in OS for young non-Hispanic Black and Hispanic women compared with non-Hispanic White women, there was no association between race and ethnicity and RFS in these groups. One possible explanation for this may be related to comorbidities: young BC survivors, who typically have longer life expectancies than older survivors if cured from BC, are at increased risk of developing cardiac toxic effects, depression, and secondary cancers.^47^ Disparities in treatment of these conditions, as well as other non–cancer-related comorbidities, may be driving the racial and ethnic disparities in OS but not RFS in younger patients. Additionally, while sample size was small, we observed that among older participants with HR+/*ERBB2*− BCs, Hispanic participants had more than 6 times worse RFS than non-Hispanic White participants. Both extremes of age and Hispanic ethnicity have been associated with reduced adherence to endocrine therapy, which may be a contributor.^18^

It is unclear why Hispanic participants in this study had significantly worse RFS than non-Hispanic White participants if they had overweight, but there was no such association in patients with obesity. Obesity has been associated with increased BC mortality in several studies, although more recent studies suggest that this association holds in White women but not in Black women.^24,25,48,49,50,51^ This is consistent with our data in the clinical trial setting and suggests that differences in obesity rates by race likely do not account for racial and ethnic disparities in BC survival.^52^ Of note, Hispanic ethnicity is underreported and underrepresented in clinical trials, and future studies with improved inclusion of Hispanic patients are necessary to confirm these findings.^26,53^

The strengths of this pooled analysis of clinical trials, including prospective collection of survival data with long follow-up, are particularly important given the outcomes of RFS and OS. Within each clinical trial, participants received similar initial treatment, and outcomes have been well ascertained. In addition to adjustment for baseline clinicopathologic risk factors, our analysis was adjusted for chemotherapy treatment received, thereby facilitating study of other factors associated with survival. By studying racial and ethnic disparities in survival in the clinical trial setting, which standardized initial care and treatment, our results emphasize that other factors, such as inequities in subsequent treatment, survivorship care, or biological differences, may contribute to these disparities. As such, this study extends the understanding of potential contributors to disparities in BC survival. We hope this will inform ongoing efforts to mitigate disparities in BC survival, such as patient navigation programs and the GETSET study^54^ (NCT04379570), as well as motivate the development of future interventions aimed at improving racial and ethnic inequities in long-term care after BC.^55,56^

### Limitations

This study has limitations. Despite the pooled nature of this analysis, there are small sample sizes within certain strata. For example, small numbers of very young patients precluded our ability to specifically study women under the age of 40 years, a group that has been shown to exhibit differences in tumor genomics and worse BC outcomes. It is possible that such biologic heterogeneity affected outcomes in the group of women younger than 50 years.^17,57,58,59^ The small sample sizes within certain subgroups, such as Hispanic participants and those with underweight BMI, likely impacts the precision of our findings. To mitigate this risk and limit the number of statistical comparisons made, we provide *P* values only for global tests comparing the main variables (race and ethnicity, age, tumor subtype, and BMI) rather than comparison of the many different levels of a variable. In the present study, it is possible that the global race and ethnicity variable was not significantly associated with RFS within any subtype due to sample size or the relatively low proportion of non-Hispanic Black and Hispanic participants, populations that are underrepresented in oncology clinical trials.^26,60,61^ Importantly, in this and many other ways, the clinical trial population does not represent typical clinical settings, and this may affect the generalizability of these data. Additionally, there was no adjustment made for multiple comparisons other than using a global test for the main variables of interest. However, throughout the manuscript, we report the testing that was done and provide estimates of observed effects.

Information on cause of death could not be reliably ascertained from all 4 of the included studies and therefore we were unable to examine differences in BC-specific survival. Additionally, as specified previously, there is not documentation of how race and ethnicity were collected for participants in every trial, which may have led to misclassification of race and ethnicity in some participants.

## Conclusions

In this cohort study of clinical trial participants treated for eBC, we observed worse survival among Black or Hispanic participants within subgroups defined by age, BMI, or tumor subtype. These data suggest that, in addition to addressing the social and structural factors that contribute to racial and ethnic disparities overall, it may be necessary to identify and address subgroup-specific mechanisms underlying the observed associations. It is critical to evaluate specific contributors to racial and ethnic disparities in survival as these may inform future interventions to improve these disparities.

## Notes

Supplement 1. **eMethods.** Supplemental Methods **eFigure 1.** Kaplan-Meier Estimates of Recurrence-Free Survival in Breast Cancer Subtype by Race and Ethnicity **eFigure 2.** Kaplan-Meier Estimates of Overall Survival in Breast Cancer Subtype by Race and Ethnicity **eFigure 3.** Kaplan-Meier Estimates of Recurrence-Free Survival in Age Category by Race and Ethnicity **eFigure 4.** Kaplan-Meier Estimates of Overall Survival in Age Category by Race and Ethnicity **eFigure 5.** Kaplan-Meier Estimates of Recurrence-Free Survival in Body Mass Index (BMI) Category by Race and Ethnicity **eFigure 6.** Kaplan-Meier Estimates of Overall Survival in Body Mass Index (BMI) Category by Race and Ethnicity
eReferences.

Supplement 2.
Data Sharing Statement
