# Errors in the Abstract

**DOI:** 10.1001/jamanetworkopen.2023.48174

**Published:** 2023-11-30

In the Original Investigation titled “Age, Body Mass Index, Tumor Subtype, and Racial and Ethnic Disparities in Breast Cancer Survival,”^1^ published October 25, 2023, there was an error in the article abstract. An association between body mass index between 25 and 30 and worse recurrence-free survival was among Hispanic participants, and not Black participants. This article has been corrected.^1^
