# Supplementary material for: Age, Body Mass Index, Tumor Subtype, and Racial and Ethnic Disparities in Breast Cancer Survival
Source: JAMA Netw Open. 2023 Oct 25;6(10):e2339584. doi: 10.1001/jamanetworkopen.2023.39584 (PMC10600583; doi:10.1001/jamanetworkopen.2023.39584)
Supplement: Supplement 1. — eMethods. Supplemental Methods eFigure 1. Kaplan-Meier Estimates of Recurrence-Free Survival in Breast Cancer Subtype by Race and Ethnicity eFigure 2. Kaplan-Meier Estimates of Overall Survival in Breast Cancer Subtype by Race and Ethnicity eFigure 3. Kaplan-Meier Estimates of Recurrence-Free Survival in Age Category by Race and Ethnicity eFigure 4. Kaplan-Meier Estimates of Overall Survival in Age Category by Race and Ethnicity eFigure 5. Kaplan-Meier Estimates of Recurrence-Free Survival in Body Mass Index (BMI) Category by Race and Ethnicity eFigure 6. Kaplan-Meier Estimates of Overall Survival in Body Mass Index (BMI) Category by Race and Ethnicity eReferences. [file jamanetwopen-e2339584-s001.pdf]

Supplemental Online Content

Lipsyc-Sharf M, Ballman KV, Campbell JD, et al. Age, body mass index, tumor subtype, and racial and ethnic disparities in breast cancer survival. *JAMA Netw Open*. 2023;6(10):e2339584. doi:10.1001/jamanetworkopen.2023.39584

eMethods. Supplemental Methods

eFigure 1. Kaplan-Meier Estimates of Recurrence-Free Survival in Breast Cancer Subtype by Race and Ethnicity

eFigure 2. Kaplan-Meier Estimates of Overall Survival in Breast Cancer Subtype by Race and Ethnicity

eFigure 3. Kaplan-Meier Estimates of Recurrence-Free Survival in Age Category by Race and Ethnicity

eFigure 4. Kaplan-Meier Estimates of Overall Survival in Age Category by Race and Ethnicity

eFigure 5. Kaplan-Meier Estimates of Recurrence-Free Survival in Body Mass Index (BMI) Category by Race and Ethnicity

eFigure 6. Kaplan-Meier Estimates of Overall Survival in Body Mass Index (BMI) Category by Race and Ethnicity

eReferences.

This supplemental material has been provided by the authors to give readers additional information about their work.

© 2023 Lipsyc-Sharf M et al. *JAMA Network Open.*

eMethods. Supplemental Methods

Pooled data from four clinical trials were included in this analysis:
Cancer and Leukemia Group B (CALGB) C9741, C49907, C40101, or North Central Cancer Treatment Group (NCCTG) N9831. Here we have included a short description of each of these clinical trials. Of note, all patients in these trials received chemotherapy. None of the patients with triple negative breast cancer in these trials received immunotherapy. Given the timing of these trials, administration of anti-HER2 therapy for patients with HER2-positive breast cancer was variable except in N9831 in which all patients received HER2-directed therapy. For additional details, please consult the full publications of these clinical trials.

CALGB C9741: This trial studied the efficacy and safety of sequential versus concurrent combination chemotherapy as well as the safety and efficacy of dose density of chemotherapy. Specifically, the four arms of the study included 1) sequential doxorubicin then paclitaxel and then cyclophosphamide, administered every 3 weeks; 2) sequential doxorubicin then paclitaxel and then cyclophosphamide, administered every 2 weeks; 3) concurrent doxorubicin and cyclophosphamide followed by paclitaxel, administered every 3 weeks; and 4) concurrent doxorubicin and cyclophosphamide followed by paclitaxel, administered every 2 weeks. The trial found that dose density improved disease-free survival (DFS) and overall survival (OS), and that there was no difference in DFS or OS between sequential and concurrent chemotherapy schedules.

CALGB C49907: This trial studied standard adjuvant chemotherapy regimens versus capecitabine in women ages 65 years and older with early-stage breast cancer and found that recurrence-free survival was significantly longer for patients receiving standard adjuvant chemotherapy than for patients receiving capecitabine. Patients that received standard chemotherapy received either CMF (cyclophosphamide, methotrexate, and fluorouracil) or AC (doxorubicin and cyclophosphamide).

CALGB C40101: This trial studied whether treatment with paclitaxel alone was non-inferior to treatment with doxorubicin and cyclophosphamide (AC) in adjuvant treatment of patients with 0 to 3 positive axillary lymph nodes. The trial found that, while paclitaxel was better tolerated than AC, the efficacy of paclitaxel was not non-inferior to AC.

NCCTG N9831: This trial studied trastuzumab administered either sequentially or concurrently with chemotherapy in adjuvant treatment of patients with early-stage HER2+ breast cancer and found that trastuzumab given concurrently with taxane chemotherapy improved DFS. Chemotherapy included treatment with doxorubicin and cyclophosphamide (AC) followed by either paclitaxel alone or paclitaxel plus trastuzumab.

© 2023 Lipsyc-Sharf M et al. *JAMA Network Open.*

eFigure 1. Kaplan-Meier Estimates of Recurrence-Free Survival in Breast Cancer Subtype by Race and Ethnicity

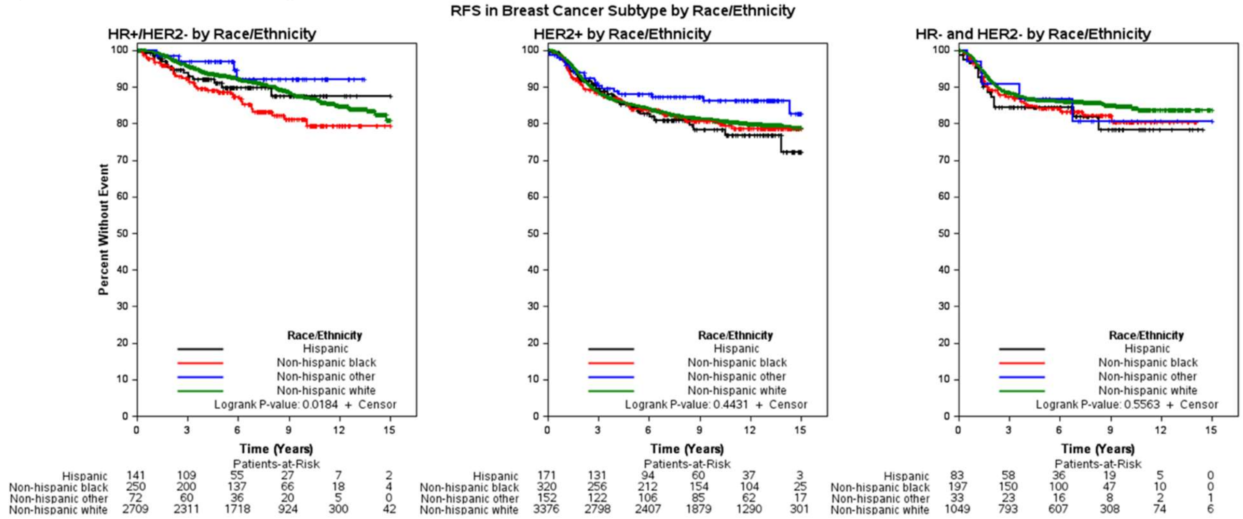

	Event/Total	Median (95% CI)[1]	Hazard Ratio (95% CI)[2]	Survival Estimates (95% CI)[1]	Covariate Level P-values	P-value
**HR+/HER2- by Race/Ethnicity**						
**Race/Ethnicity**						0.0184[3]
Hispanic	13/141	NE (NE-NE)	1.17 (0.67-2.05)	5 Years: 91.0 (86.1-96.3%)	0.5721[4]	
				10 Years: 87.5 (80.6-94.9%)		
Non-hispanic black	37/250	NE (NE-NE)	1.67 (1.18-2.35)	5 Years: 88.5 (84.5-92.8%)	0.0035[4]	
				10 Years: 81.0 (75.3-87.2%)		
Non-hispanic other	4/72	NE (NE-NE)	0.62 (0.23-1.67)	5 Years: 97.0 (93.0-100.0%)	0.3464[4]	
				10 Years: 92.1 (84.8-100.0%)		
Non-hispanic white	269/2709	NE (NE-NE)	Reference	5 Years: 93.2 (92.2-94.2%)	--	
				10 Years: 87.2 (85.7-88.8%)		
**HER2+ by Race/Ethnicity**						
**Race/Ethnicity**						0.4431[3]
Hispanic	32/171	NE (NE-NE)	1.14 (0.80-1.62)	5 Years: 85.1 (79.6-91.0%)	0.4740[4]	
				10 Years: 78.4 (71.6-85.9%)		
Non-hispanic black	59/320	NE (NE-NE)	1.05 (0.81-1.38)	5 Years: 84.0 (79.9-88.3%)	0.7012[4]	
				10 Years: 80.5 (76.0-85.3%)		
Non-hispanic other	20/152	NE (NE-NE)	0.73 (0.47-1.14)	5 Years: 88.0 (82.8-93.5%)	0.1687[4]	
				10 Years: 86.1 (80.4-92.2%)		
Non-hispanic white	621/3376	NE (NE-NE)	Reference	5 Years: 85.1 (83.8-86.3%)	--	
				10 Years: 80.8 (79.4-82.2%)		
**HR- and HER2- by Race/Ethnicity**						
**Race/Ethnicity**						0.5563[3]
Hispanic	14/83	NE (NE-NE)	1.35 (0.78-2.33)	5 Years: 84.6 (76.9-93.0%)	0.2886[4]	
				10 Years: 78.3 (67.7-90.5%)		
Non-hispanic black	32/197	NE (NE-NE)	1.23 (0.84-1.81)	5 Years: 84.1 (79.0-89.6%)	0.2862[4]	
				10 Years: 80.4 (73.9-87.4%)		
Non-hispanic other	5/33	NE (NE-NE)	1.19 (0.49-2.90)	5 Years: 86.8 (75.4-99.9%)	0.7041[4]	
				10 Years: 80.6 (65.8-98.6%)		
Non-hispanic white	142/1049	NE (NE-NE)	Reference	5 Years: 86.6 (84.5-88.8%)	--	
				10 Years: 84.7 (82.3-87.2%)		

[1]Kaplan-Meier method; [2]Cox model; [3]Logrank test; [4]Wald Chi-Square test;

These supplemental Kaplan-Meier estimates show unadjusted survival by subgroup.

Abbreviations: RFS, recurrence-free survival; HR+, hormone receptor positive; HER2-, human epidermal growth factor receptor 2-negative; HER2+, human epidermal growth factor receptor 2-positive; HR-, hormone receptor negative.

© 2023 Lipsyc-Sharf M et al. *JAMA Network Open.*

eFigure 2. Kaplan-Meier Estimates of Overall Survival in Breast Cancer Subtype by Race and Ethnicity

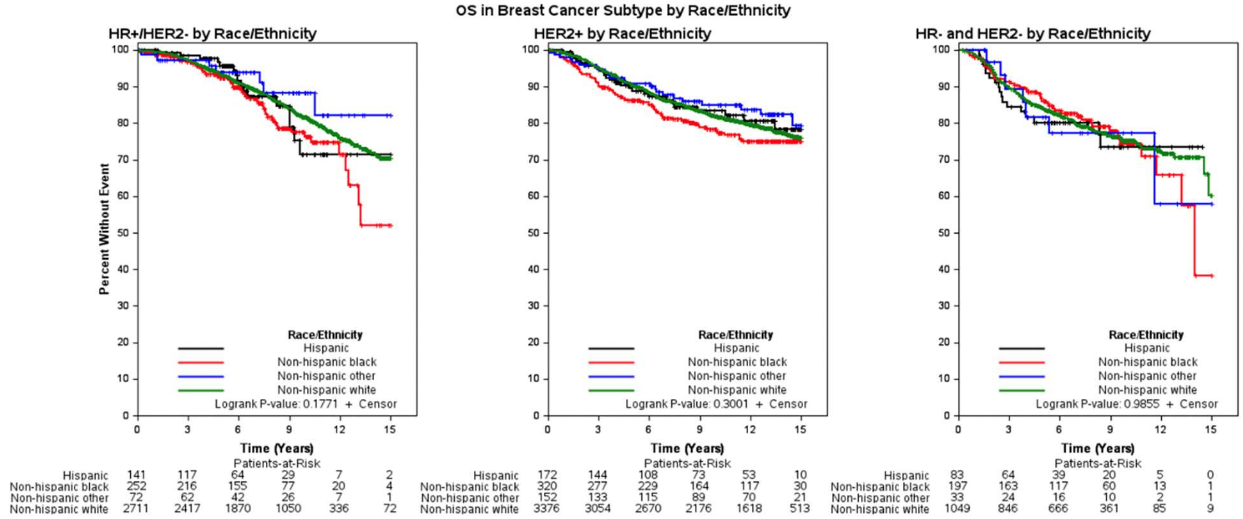

	Event/Total	Median (95% CI)[1]	Hazard Ratio (95% CI)[2]	Survival Estimates (95% CI)[1]	Covariate Level P-values	P-value
**HR+/HER2- by Race/Ethnicity**						
**Race/Ethnicity**						0.1771[3]
Hispanic	16/141	NE (NE-NE)	1.06 (0.64-1.75)	5 Years: 95.7 (92.0-99.5%)	0.8154[4]	
				10 Years: 71.4 (58.5-87.0%)		
Non-hispanic black	46/252	NE (13.1-NE)	1.36 (1.01-1.85)	5 Years: 92.4 (89.0-96.0%)	0.0458[4]	
				10 Years: 76.2 (69.7-83.4%)		
Non-hispanic other	7/72	NE (NE-NE)	0.72 (0.34-1.52)	5 Years: 93.8 (88.1-99.9%)	0.3883[4]	
				10 Years: 88.4 (79.6-98.1%)		
Non-hispanic white	411/2711	NE (NE-NE)	Reference	5 Years: 93.2 (92.2-94.2%)	--	
				10 Years: 81.6 (79.7-83.4%)		
**HER2+ by Race/Ethnicity**						
**Race/Ethnicity**						0.3001[3]
Hispanic	26/172	NE (NE-NE)	0.95 (0.64-1.40)	5 Years: 88.8 (83.9-94.0%)	0.7906[4]	
				10 Years: 83.3 (77.2-90.0%)		
Non-hispanic black	68/320	NE (NE-NE)	1.22 (0.95-1.57)	5 Years: 86.3 (82.6-90.3%)	0.1147[4]	
				10 Years: 77.8 (73.0-82.9%)		
Non-hispanic other	23/152	NE (NE-NE)	0.82 (0.54-1.24)	5 Years: 90.9 (86.3-95.7%)	0.3489[4]	
				10 Years: 84.9 (79.0-91.3%)		
Non-hispanic white	659/3376	NE (NE-NE)	Reference	5 Years: 90.5 (89.5-91.5%)	--	
				10 Years: 81.8 (80.5-83.2%)		
**HR- and HER2- by Race/Ethnicity**						
**Race/Ethnicity**						0.9855[3]
Hispanic	17/83	NE (NE-NE)	1.08 (0.66-1.77)	5 Years: 80.2 (71.7-89.7%)	0.7557[4]	
				10 Years: 73.5 (62.4-86.7%)		
Non-hispanic black	41/197	14.0 (13.2-NE)	1.02 (0.73-1.42)	5 Years: 86.1 (81.1-91.3%)	0.9215[4]	
				10 Years: 74.3 (66.7-82.8%)		
Non-hispanic other	7/33	NE (11.6-NE)	1.09 (0.52-2.32)	5 Years: 81.7 (68.4-97.6%)	0.8146[4]	
				10 Years: 77.2 (62.6-95.3%)		
Non-hispanic white	222/1049	NE (14.9-NE)	Reference	5 Years: 84.2 (81.9-86.5%)	--	
				10 Years: 75.4 (72.4-78.5%)		

[1]Kaplan-Meier method; [2]Cox model; [3]Logrank test; [4]Wald Chi-Square test;

These supplemental Kaplan-Meier estimates show unadjusted survival by subgroup.

Abbreviations: OS, overall survival; HR+, hormone receptor positive; HER2-, human epidermal growth factor receptor 2-negative; HER2+, human epidermal growth factor receptor 2-positive; HR-, hormone receptor negative.

© 2023 Lipsyc-Sharf M et al. *JAMA Network Open.*

eFigure 3. Kaplan-Meier Estimates of Recurrence-Free Survival in Age Category by Race and Ethnicity

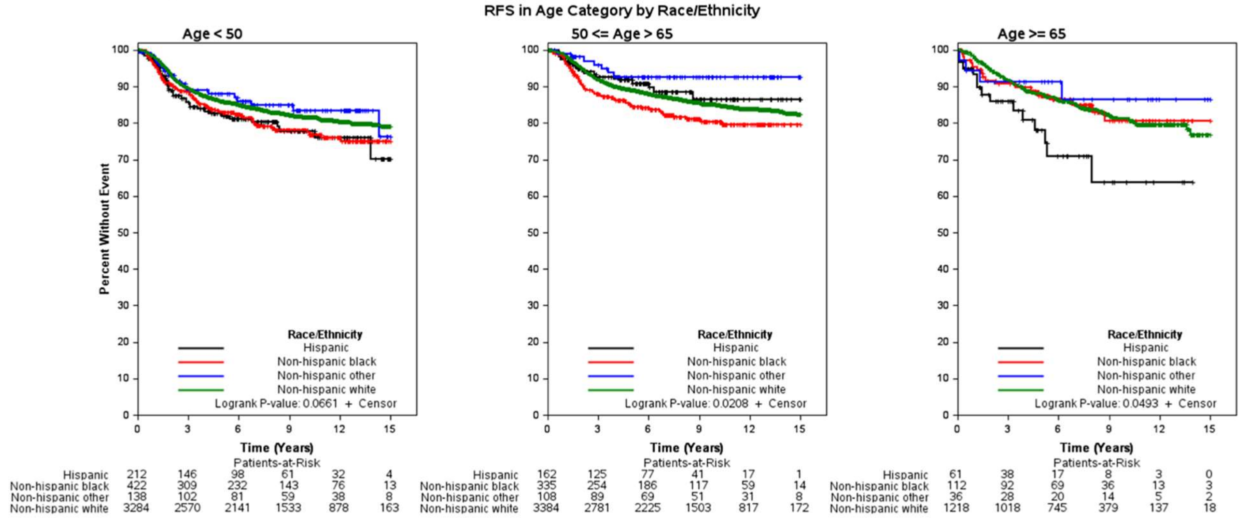

	Event/Total	Median (95% CI)[1]	Hazard Ratio (95% CI)[2]	Survival Estimates (95% CI)[1]	Covariate Level P-values	P-value
**Age < 50**						
**Race/Ethnicity**						0.0661[3]
Hispanic	40/212	NE (NE-NE)	1.32 (0.96-1.82)	5 Years: 82.6 (77.4-88.2%)	0.0875[4]	
				10 Years: 77.8 (71.4-84.7%)		
Non-hispanic black	81/422	NE (NE-NE)	1.29 (1.02-1.63)	5 Years: 82.8 (79.0-86.7%)	0.0337[4]	
				10 Years: 78.1 (73.8-82.7%)		
Non-hispanic other	20/138	NE (NE-NE)	0.92 (0.59-1.43)	5 Years: 88.1 (82.6-94.0%)	0.6971[4]	
				10 Years: 83.3 (76.5-90.7%)		
Non-hispanic white	542/3284	NE (NE-NE)	Reference	5 Years: 85.9 (84.7-87.2%)	--	
				10 Years: 81.7 (80.2-83.1%)		
**50 <= Age > 65**						
**Race/Ethnicity**						0.0208[3]
Hispanic	16/162	NE (NE-NE)	0.84 (0.51-1.39)	5 Years: 91.8 (87.5-96.4%)	0.5049[4]	
				10 Years: 86.6 (80.1-93.5%)		
Non-hispanic black	56/335	NE (NE-NE)	1.37 (1.04-1.81)	5 Years: 84.6 (80.6-88.7%)	0.0266[4]	
				10 Years: 80.4 (75.7-85.4%)		
Non-hispanic other	7/108	NE (NE-NE)	0.48 (0.23-1.02)	5 Years: 92.7 (87.6-98.1%)	0.0569[4]	
				10 Years: 92.7 (87.6-98.1%)		
Non-hispanic white	452/3384	NE (NE-NE)	Reference	5 Years: 89.1 (88.0-90.2%)	--	
				10 Years: 85.0 (83.7-86.3%)		
**Age >= 65**						
**Race/Ethnicity**						0.0493[3]
Hispanic	14/61	NE (8.0-NE)	2.09 (1.21-3.60)	5 Years: 78.0 (66.9-90.8%)	0.0080[4]	
				10 Years: 63.8 (47.7-85.1%)		
Non-hispanic black	17/112	NE (NE-NE)	0.99 (0.60-1.63)	5 Years: 87.6 (81.5-94.2%)	0.9734[4]	
				10 Years: 80.7 (72.3-90.1%)		
Non-hispanic other	4/36	NE (NE-NE)	0.75 (0.28-2.03)	5 Years: 91.2 (82.2-100.0%)	0.5768[4]	
				10 Years: 86.4 (74.5-100.0%)		
Non-hispanic white	190/1218	NE (NE-NE)	Reference	5 Years: 87.9 (86.0-89.8%)	--	
				10 Years: 81.0 (78.4-83.7%)		

[1]Kaplan-Meier method; [2]Cox model; [3]Logrank test; [4]Wald Chi-Square test;

These supplemental Kaplan-Meier estimates show unadjusted survival by subgroup.
Age is represented in years.
Abbreviations: RFS, recurrence-free survival.

© 2023 Lipsyc-Sharf M et al. *JAMA Network Open.*

eFigure 4. Kaplan-Meier Estimates of Overall Survival in Age Category by Race and Ethnicity

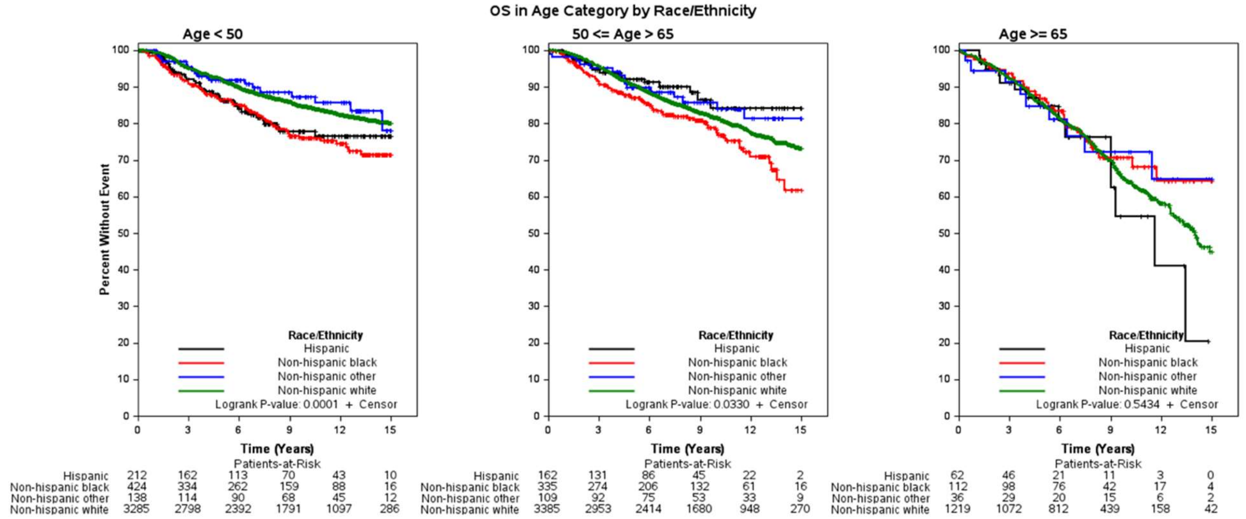

	Event/Total	Median (95% CI)[1]	Hazard Ratio (95% CI)[2]	Survival Estimates (95% CI)[1]	Covariate Level P-values	P-value
**Age < 50**						
**Race/Ethnicity**						0.0001[3]
Hispanic	36/212	NE (NE-NE)	1.46 (1.04-2.06)	5 Years: 86.2 (81.2-91.4%)	0.0275[4]	
				10 Years: 77.9 (71.5-84.9%)		
Non-hispanic black	84/424	NE (NE-NE)	1.62 (1.28-2.04)	5 Years: 86.6 (83.2-90.1%)	<0.0001[4]	
				10 Years: 75.9 (71.2-80.9%)		
Non-hispanic other	17/138	NE (NE-NE)	0.91 (0.56-1.48)	5 Years: 91.9 (87.2-96.9%)	0.7044[4]	
				10 Years: 87.3 (81.2-93.9%)		
Non-hispanic white	475/3285	NE (NE-NE)	Reference	5 Years: 92.0 (91.0-92.9%)	--	
				10 Years: 84.6 (83.3-86.0%)		
**50 <= Age > 65**						
**Race/Ethnicity**						0.0330[3]
Hispanic	16/162	NE (NE-NE)	0.72 (0.44-1.18)	5 Years: 92.2 (87.8-96.8%)	0.1943[4]	
				10 Years: 84.3 (76.6-92.8%)		
Non-hispanic black	70/335	NE (NE-NE)	1.35 (1.05-1.73)	5 Years: 87.0 (83.3-90.8%)	0.0179[4]	
				10 Years: 77.5 (72.4-83.1%)		
Non-hispanic other	15/109	NE (NE-NE)	0.78 (0.47-1.31)	5 Years: 89.7 (83.8-96.0%)	0.3536[4]	
				10 Years: 85.7 (78.7-93.3%)		
Non-hispanic white	606/3385	NE (NE-NE)	Reference	5 Years: 90.6 (89.5-91.6%)	--	
				10 Years: 81.5 (80.0-83.0%)		
**Age >= 65**						
**Race/Ethnicity**						0.5434[3]
Hispanic	15/62	11.6 (9.0-NE)	1.20 (0.72-2.02)	5 Years: 84.6 (75.2-95.2%)	0.4860[4]	
				10 Years: 54.6 (35.7-83.6%)		
Non-hispanic black	29/112	NE (NE-NE)	0.82 (0.56-1.19)	5 Years: 86.7 (80.4-93.5%)	0.2959[4]	
				10 Years: 70.7 (61.7-80.9%)		
Non-hispanic other	9/36	NE (11.5-NE)	0.79 (0.41-1.52)	5 Years: 84.8 (73.3-98.1%)	0.4772[4]	
				10 Years: 72.1 (57.0-91.2%)		
Non-hispanic white	385/1219	14.0 (13.0-NE)	Reference	5 Years: 85.3 (83.3-87.4%)	--	
				10 Years: 64.4 (61.2-67.7%)		

[1]Kaplan-Meier method; [2]Cox model; [3]Logrank test; [4]Wald Chi-Square test;

These supplemental Kaplan-Meier estimates show unadjusted survival by subgroup.
Age is represented in years.
Abbreviation: RFS, recurrence-free survival.

© 2023 Lipsyc-Sharf M et al. *JAMA Network Open.*

eFigure 5. Kaplan-Meier Estimates of Recurrence-Free Survival in Body Mass Index (BMI) Category by Race and Ethnicity

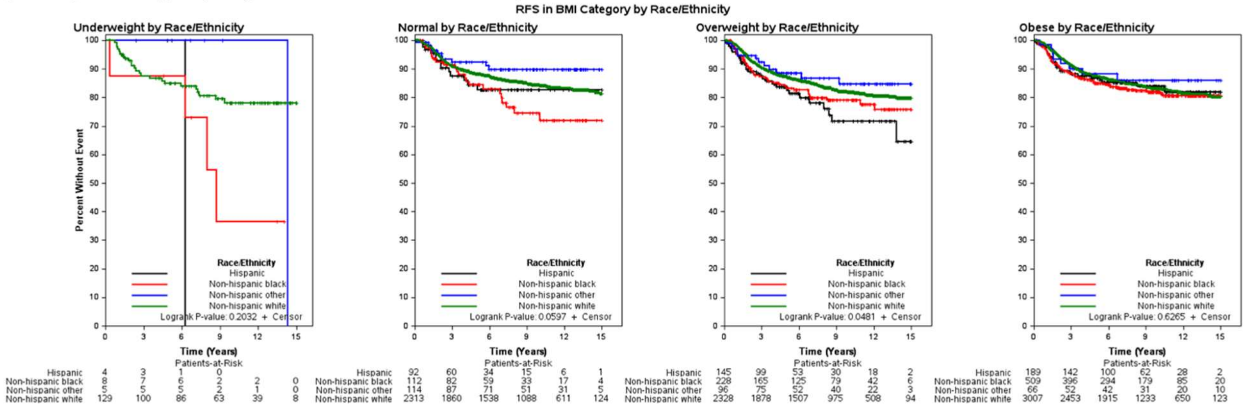

Body Mass Index (BMI) groups were determined based on National Institutes of Health definitions of underweight, normal weight, overweight, and obesity as follows: <18.5, 18.5 to <25, 25 to <30, ≥30 kg/m^2 respectively.[24]

	Event/Total	Median (95% CI)[1]	Hazard Ratio (95% CI)[2]	Survival Estimates (95% CI)[1]	Covariate Level P-values	P-value
**Underweight by Race/Ethnicity**						
**Race/Ethnicity**						0.2032[3]
Hispanic	1/4	6.3 (NE-NE)	1.93 (0.26-14.52)	5 Years: 100.0 (100.0-100.0%)	0.5224[4]	
				10 Years: 0.0 (NE-NE%)		
Non-hispanic black	4/8	8.7 (6.3-NE)	2.93 (1.01-8.50)	5 Years: 87.5 (67.3-100.0%)	0.0471[4]	
				10 Years: 36.5 (12.4-100.0%)		
Non-hispanic other	1/5	14.3 (NE-NE)	0.97 (0.13-7.21)	5 Years: 100.0 (100.0-100.0%)	0.9797[4]	
				10 Years: 100.0 (100.0-100.0%)		
Non-hispanic white	24/129	NE (NE-NE)	Reference	5 Years: 84.9 (78.7-91.6%)	--	
				10 Years: 78.2 (70.7-86.5%)		
**Normal by Race/Ethnicity**						
**Race/Ethnicity**						0.0597[3]
Hispanic	13/92	NE (NE-NE)	1.28 (0.74-2.24)	5 Years: 84.4 (76.5-93.0%)	0.3784[4]	
				10 Years: 82.5 (74.2-91.8%)		
Non-hispanic black	22/112	NE (NE-NE)	1.59 (1.03-2.45)	5 Years: 84.4 (77.3-92.0%)	0.0355[4]	
				10 Years: 74.4 (65.1-85.1%)		
Non-hispanic other	10/114	NE (NE-NE)	0.64 (0.34-1.20)	5 Years: 92.3 (87.3-97.6%)	0.1672[4]	
				10 Years: 89.8 (84.0-96.1%)		
Non-hispanic white	327/2313	NE (NE-NE)	Reference	5 Years: 88.3 (86.9-89.7%)	--	
				10 Years: 84.3 (82.7-85.9%)		
**Overweight by Race/Ethnicity**						
**Race/Ethnicity**						0.0481[3]
Hispanic	29/145	NE (NE-NE)	1.56 (1.07-2.28)	5 Years: 83.7 (77.5-90.4%)	0.0212[4]	
				10 Years: 71.6 (62.1-82.6%)		
Non-hispanic black	42/228	NE (NE-NE)	1.29 (0.94-1.77)	5 Years: 83.2 (78.1-88.6%)	0.1215[4]	
				10 Years: 79.0 (73.3-85.2%)		
Non-hispanic other	12/96	NE (NE-NE)	0.83 (0.47-1.48)	5 Years: 88.6 (82.1-95.5%)	0.5312[4]	
				10 Years: 84.6 (76.6-93.4%)		
Non-hispanic white	368/2328	NE (NE-NE)	Reference	5 Years: 87.3 (85.9-88.7%)	--	
				10 Years: 82.0 (80.2-83.7%)		
**Obese by Race/Ethnicity**						
**Race/Ethnicity**						0.6265[3]
Hispanic	27/189	NE (NE-NE)	1.06 (0.72-1.57)	5 Years: 86.8 (81.8-92.0%)	0.7608[4]	
				10 Years: 83.9 (78.2-90.0%)		
Non-hispanic black	82/509	NE (NE-NE)	1.15 (0.91-1.45)	5 Years: 84.9 (81.7-88.2%)	0.2512[4]	
				10 Years: 81.9 (78.3-85.7%)		
Non-hispanic other	8/66	NE (NE-NE)	0.82 (0.41-1.65)	5 Years: 88.2 (80.3-96.8%)	0.5781[4]	
				10 Years: 86.1 (77.5-95.6%)		
Non-hispanic white	449/3007	NE (NE-NE)	Reference	5 Years: 87.5 (86.2-88.7%)	--	
				10 Years: 83.2 (81.7-84.7%)		

[1]Kaplan-Meier method; [2]Cox model; [3]Logrank test; [4]Wald Chi-Square test;

These supplemental Kaplan-Meier estimates show unadjusted survival by subgroup.
Abbreviations: RFS, recurrence-free survival; BMI, body mass index.

© 2023 Lipsyc-Sharf M et al. *JAMA Network Open.*

eFigure 6. Kaplan-Meier Estimates of Overall Survival in Body Mass Index (BMI) Category by Race and Ethnicity

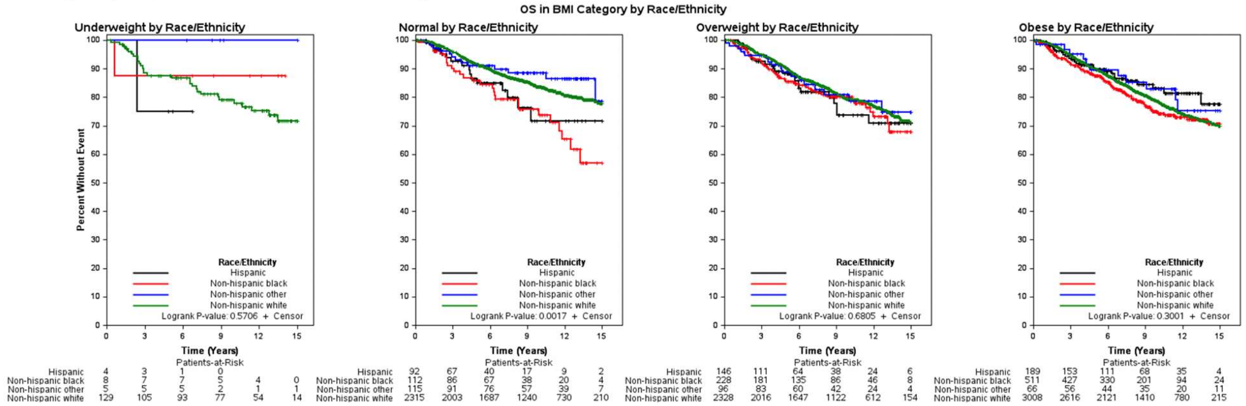

Body Mass Index (BMI) groups were determined based on National Institutes of Health definitions of underweight, normal weight, overweight, and obesity as follows: <18.5, 18.5 to <25, 25 to <30, ≥30 kg/m^2 respectively.[24]

	Event/Total	Median (95% CI)[1]	Hazard Ratio (95% CI)[2]	Survival Estimates (95% CI)[1]	Covariate Level P-values	P-value
**Underweight by Race/Ethnicity**						
**Race/Ethnicity**						0.5706[3]
Hispanic	1/4	NE (2.4-NE)	1.89 (0.25-14.18)	5 Years: 75.0 (42.6-100.0%)	0.5342[4]	
				10 Years: NE (NE-NE%)		
Non-hispanic black	1/8	NE (NE-NE)	0.53 (0.07-3.88)	5 Years: 87.5 (67.3-100.0%)	0.5312[4]	
				10 Years: 87.5 (67.3-100.0%)		
Non-hispanic other	0/5	NE (NE-NE)	0.00 (0.00-)	5 Years: 100.0 (100.0-100.0%)	0.9895[4]	
				10 Years: 100.0 (100.0-100.0%)		
Non-hispanic white	29/129	NE (NE-NE)	Reference	5 Years: 87.6 (81.9-93.7%)	--	
				10 Years: 79.0 (71.8-86.9%)		
**Normal by Race/Ethnicity**						
**Race/Ethnicity**						0.0017[3]
Hispanic	15/92	NE (NE-NE)	1.60 (0.95-2.69)	5 Years: 85.0 (77.1-93.7%)	0.0745[4]	
				10 Years: 71.6 (58.5-87.6%)		
Non-hispanic black	27/112	NE (12.5-NE)	1.93 (1.31-2.86)	5 Years: 85.7 (79.1-93.0%)	0.0010[4]	
				10 Years: 73.7 (64.4-84.4%)		
Non-hispanic other	13/115	NE (NE-NE)	0.78 (0.45-1.36)	5 Years: 91.1 (85.6-96.8%)	0.3786[4]	
				10 Years: 88.5 (82.3-95.2%)		
Non-hispanic white	359/2315	NE (NE-NE)	Reference	5 Years: 91.5 (90.3-92.7%)	--	
				10 Years: 83.6 (82.0-85.3%)		
**Overweight by Race/Ethnicity**						
**Race/Ethnicity**						0.6805[3]
Hispanic	25/146	NE (NE-NE)	1.18 (0.79-1.77)	5 Years: 88.1 (82.7-94.0%)	0.4115[4]	
				10 Years: 73.8 (64.4-84.6%)		
Non-hispanic black	44/228	NE (NE-NE)	1.16 (0.85-1.59)	5 Years: 85.4 (80.6-90.5%)	0.3344[4]	
				10 Years: 80.0 (74.4-86.1%)		
Non-hispanic other	17/96	NE (NE-NE)	1.01 (0.62-1.64)	5 Years: 88.7 (82.4-95.6%)	0.9668[4]	
				10 Years: 80.8 (72.2-90.3%)		
Non-hispanic white	447/2328	NE (NE-NE)	Reference	5 Years: 90.2 (88.9-91.4%)	--	
				10 Years: 79.3 (77.5-81.2%)		
**Obese by Race/Ethnicity**						
**Race/Ethnicity**						0.3001[3]
Hispanic	26/189	NE (NE-NE)	0.80 (0.54-1.18)	5 Years: 89.8 (85.3-94.5%)	0.2543[4]	
				10 Years: 83.1 (76.8-89.9%)		
Non-hispanic black	106/511	NE (NE-NE)	1.14 (0.93-1.40)	5 Years: 87.6 (84.7-90.6%)	0.2141[4]	
				10 Years: 74.7 (70.3-79.3%)		
Non-hispanic other	11/66	NE (NE-NE)	0.81 (0.44-1.46)	5 Years: 89.6 (82.0-97.9%)	0.4763[4]	
				10 Years: 82.8 (73.0-93.9%)		
Non-hispanic white	607/3008	NE (NE-NE)	Reference	5 Years: 89.8 (88.7-91.0%)	--	
				10 Years: 78.6 (76.9-80.3%)		

[1]Kaplan-Meier method; [2]Cox model; [3]Logrank test; [4]Wald Chi-Square test;

These supplemental Kaplan-Meier estimates show unadjusted survival by subgroup.
Abbreviations: RFS, recurrence-free survival; BMI, body mass index.

© 2023 Lipsyc-Sharf M et al. *JAMA Network Open.*

eReferences.

1. Muss HB, Berry DA, Cirrincione CT, et al. Adjuvant Chemotherapy in Older Women with Early-Stage Breast Cancer. *New England Journal of Medicine*. 2009;360(20):2055-2065. doi:10.1056/NEJMoa0810266
2. Perez EA, Suman VJ, Davidson NE, et al. Sequential versus concurrent trastuzumab in adjuvant chemotherapy for breast cancer. *J Clin Oncol*. Dec 1 2011;29(34):4491-7. doi:10.1200/jco.2011.36.7045
3. Shulman LN, Berry DA, Cirrincione CT, et al. Comparison of doxorubicin and cyclophosphamide versus single-agent paclitaxel as adjuvant therapy for breast cancer in women with 0 to 3 positive axillary nodes: CALGB 40101 (Alliance). *J Clin Oncol*. Aug 1 2014;32(22):2311-7. doi:10.1200/jco.2013.53.7142
4. Citron ML, Berry DA, Cirrincione C, et al. Randomized trial of dose-dense versus conventionally scheduled and sequential versus concurrent combination chemotherapy as postoperative adjuvant treatment of node-positive primary breast cancer: first report of Intergroup Trial C9741/Cancer and Leukemia Group B Trial 9741. *J Clin Oncol*. Apr 15 2003;21(8):1431-9. doi:10.1200/jco.2003.09.081

© 2023 Lipsyc-Sharf M et al. *JAMA Network Open.*
