# Supplementary material for: Age, Body Mass Index, Tumor Subtype, and Racial and Ethnic Disparities in Breast Cancer Survival
Source: JAMA Netw Open. 2023 Oct 25;6(10):e2339584. doi: 10.1001/jamanetworkopen.2023.39584 (PMC10600583; doi:10.1001/jamanetworkopen.2023.39584)
Supplement: Supplement 2. — Data Sharing Statement [file jamanetwopen-e2339584-s002.pdf]

Data Sharing Statement

Lipsyc-Sharf. Age, Body Mass Index, Tumor Subtype, and Racial and Ethnic Disparities in Breast Cancer Survival. *JAMA Netw Open*. Published October 25, 2023. doi:10.1001/jamanetworkopen.2023.39584

Data
Data available: No
